# Microglial Morphometric Parameters Correlate With the Expression Level of IL-1β, and Allow Identifying Different Activated Morphotypes

**DOI:** 10.3389/fncel.2019.00472

**Published:** 2019-10-25

**Authors:** María del Mar Fernández-Arjona, Jesús M. Grondona, Pedro Fernández-Llebrez, María D. López-Ávalos

**Affiliations:** ^1^Departamento de Biología Celular, Genética y Fisiología, Facultad de Ciencias, Universidad de Málaga, Málaga, Spain; ^2^Instituto de Investigación Biomédica de Málaga-IBIMA, Málaga, Spain

**Keywords:** microglia, morphotypes, interleukine-1β, hierarchical cluster analysis, neuraminidase

## Abstract

Microglia are the resident macrophages in the brain. Traditionally, two forms of microglia have been described: one considered as a resting/surveillant state in which cells have a highly branched morphology, and another considered as an activated state in which they acquire a de-ramified or amoeboid form. However, many studies describe intermediate microglial morphologies which emerge during pathological processes. Since microglial form and function are closely related, it is of interest to correlate microglial morphology with the extent of its activation. To address this issue, we used a rat model of neuroinflammation consisting in a single injection of the enzyme neuraminidase (NA) within the lateral ventricle. Sections from NA-injected animals were co-immunolabeled with the microglial marker IBA1 and the cytokine IL-1β, which highlight features of the cell’s shape and inflammatory activation, respectively. Activated (IL-1β positive) microglial cells were sampled from the dorsal hypothalamus nearby the third ventricle. Images of single microglial cells were processed in two different ways to obtain (1) an accurate measure of the level of expression of IL-1β (indicating the degree of activation), and (2) a set of 15 morphological parameters to quantitatively and objectively describe the cell’s shape. A simple regression analysis revealed a dependence of most of the morphometric parameters on IL-1β expression, demonstrating that the morphology of microglial cells changes progressively with the degree of activation. Moreover, a hierarchical cluster analysis pointed out four different morphotypes of activated microglia, which are characterized not only by morphological parameters values, but also by specific IL-1β expression levels. Thus, these results demonstrate in an objective manner that the activation of microglial cells is a gradual process, and correlates with their morphological change. Even so, it is still possible to categorize activated cells according to their morphometric parameters, each category presenting a different activation degree. The physiological relevance of those activated morphotypes is an issue worth to be assessed in the future.

## Introduction

Microglial cells are the resident macrophages in the central nervous system. Initially they were considered to be “resting” or “quiescent” in normal or healthy conditions, eventually acquiring an activated phenotype in pathological situations (Davis et al., 1994). However, more recent studies have suggested that microglia play several roles both in the healthy and the pathological brain (Biber and Block, 2014). Thus, the main role of microglia in the healthy brain is to survey the nervous tissue environment, so the previously presumed resting state is actually very active, continuously monitoring the surroundings, and acting as main sensors of brain alterations (Nimmerjahn et al., 2005; Nimmerjahn, 2012). Besides, microglial cells carry out diverse maintenance tasks to provide a propitious physiological environment (Gomez-Nicola and Perry, 2015). In addition, under various stimulus (e.g., infection, trauma or stroke) microglia may acquire different activated phenotypes to engage immunological or repair functions (Ransohoff and Perry, 2009). Those activated phenotypes have been defined as classical activation (M1), alternative activation (M2a), type II alternative activation (M2b), and acquired deactivation (M2c). Among them, M1 is considered a pro-inflammatory phenotype, while M2 subtypes are considered anti-inflammatory states. However, this classification is highly controversial, and many authors do not agree with it. In fact, this scenario is more intricate, since microglial cells exhibit a great plasticity within each specialized niche. Microglia phenotype is usually not absolute, but may display features of different states, or even a continuum of various activated states (Olah et al., 2011; Ransohoff, 2016). In addition, microglial cells can play a confusing toxic or protective role in particular neurological pathologies (Biber and Block, 2014), which represents a crucial issue to unravel.

It is widely accepted that the function of microglial cells and their form are closely coupled (Davis et al., 1994). Therefore, the morphological analysis by quantitative and objective methods is considered as a valuable tool to better understand form and function relationships. In addition, because microglial cells are highly dynamic, quantitative analysis could detect subtle changes in cell morphology. Numerous studies have addressed the morphological analysis of microglia, although many of them used qualitative and subjective approaches to classify them. During the last decade the use of quantitative objective methods to study morphology has gained interest. In this way, microglial cells have been analyzed in healthy brains of both humans and rats (Kongsui et al., 2014; Torres-Platas et al., 2014) as well as in different pathological situations such as brain injuries (Soltys et al., 2001; Zanier et al., 2015), under physiological (salt load) or psychological stress (Ayoub and Salm, 2003; Hinwood et al., 2013), and in mouse models of Alzheimer’s disease (Baron et al., 2014). Some studies tried to establish different microglial morphotypes based on morphological parameters. Statistical procedures such as principal component analysis revealed the morphological parameters with greater discriminant capacity (Soltys et al., 2005). Hierarchical cluster analysis, which allows grouping objects based on their similarity, was used to suggest new microglia classifications in different experimental models, such as visual learning in monkeys (Santos-Filho et al., 2014), hypoglossal axotomy (Yamada and Jinno, 2013), experimental neuroinflammation (de Sousa et al., 2015), and a mouse model of amyotrophic lateral sclerosis (Ohgomori et al., 2016). These works objectively proposed different morphotypes, which have been associated to a particular physiological/pathological spatiotemporal condition.

The current study has focused on the morphology of hypothalamic microglia undergoing inflammatory activation. To induce the activation of these cells, an acute neuroinflammation was generated by the intracerebroventricular administration of microbial neuraminidase (Grondona et al., 1996; Del Carmen Gómez-Roldán et al., 2008; Granados-Durán et al., 2015). Neuraminidase (NA) is a sialidase enzyme that removes terminal sialic acid from the carbohydrate chains of glycoproteins and glycolipids. When NA is injected into the lateral ventricle of rodents, it is distributed by the flow of cerebrospinal fluid, affecting the ventricular system and periventricular regions, including the hypothalamus. The diffusion of the enzyme from the ventricular cavity toward the hypothalamic parenchyma provokes the inflammatory activation of resident microglia (Granados-Durán et al., 2015; Fernández-Arjona et al., 2017). On the basis of immunostaining for the pro-inflammatory cytokine IL-1β, the level of activation of these hypothalamic microglial cells appears to be heterogeneous, as well as their morphology. Hence, we tried to correlate the level of activation (assessed by IL-1β immunostaining) with different morphometric parameters measured in single microglial cells. Also, we aimed to defining different subgroups of activated microglia based on the objective quantification of morphological parameters. Our results point that most of the morphometric parameters measured show a dependence on the level of expression of IL-1β, and they progressively change as the cell becomes more activated. Even though the morphological change that accompanies activation is gradual, with the aid of statistical methods it is possible to categorize activated microglial cells using specific morphological parameters. The morphology-based classification obtained is in accordance with different IL-1β expression levels, indicating the value of morphological analysis to examine the degree of inflammatory activation of microglial cells.

## Materials and Methods

### Animals

Six male Wistar rats (350 g, about 10 weeks old) were provided by Charles River Laboratories (Barcelona, Spain). Animals were maintained in the animal house at Universidad de Málaga, under a 12 h light/dark cycle, at 23°C and 60% humidity, with food and water available *ad libitum*. Animal care and handling were performed according to the guidelines established by the Spanish legislation (RD 53/2013) and the European Union regulation (2010/63/EU). All procedures performed were approved by the ethics committee of Universidad de Málaga (Comité Ético de Experimentación de la Universidad de Málaga; reference 2012-0013). All efforts were made to minimize the number of animals used and their suffering.

### Intracerebroventricular Injection

An acute and sterile neuroinflammatory process was generated in rats by a single injection of the enzyme NA within the right lateral ventricle of the brain (Grondona et al., 1996; Del Carmen Gómez-Roldán et al., 2008; Granados-Durán et al., 2015). Intracerebroventricular (ICV) injection procedure was performed as previously described (Granados-Durán et al., 2015). Briefly, the animals were anesthetized with ketamine/xylazine solution (80 and 12 mg/kg, respectively; Sigma-Aldrich) and positioned in a stereotaxic frame. A scalp incision along the sagittal midline was performed to access the skull and the bone was perforated with a drill in the following coordinates: 0.5 mm posterior, and 1.4 mm lateral from Bregma (Paxinos and Watson, 2007). Neuraminidase from *Clostridium perfringens* (Sigma-Aldrich, N3001) dissolved in 0.9% sterile saline was administered by a single injection 3.5 mm below the dura mater, with the aid of a pump; a dose of 500 mU (in 20 μL) of NA was perfused during 10 min at a rate of 2 μL/min. The animals were sacrificed at 12 h post-injection. Sham (saline-injected) animals were not included, because: (1) from previous studies (Fernández-Arjona et al., 2017) we knew that IL-1β expression in hypothalamic microglial cells was absent, (2) the aim of this study was focused on activated microglial cells, and (3) in case we wanted to sample IL-1β negative cells, it would be possible to find them in regions of the brain parenchyma farther from the ventricular surface.

### Brain Sections and Immunohistochemistry

Prior to sacrifice, the animals were anesthetized (as described above) and systemically perfused with 0.9% saline, followed by 4% parafolmaldehyde. Brains were removed and post-fixed overnight in the same fixative solution. Free floating coronal sections of brain tissue were later obtained with a vibratome (40 μm thickness), and the sections were cryoprotected with a sucrose and ethylene glycol solution (30% w/v and 30% v/v respectively, in 0.1 M phosphate buffer). Brain sections including the third ventricle (distance from Bregma about −3.30 mm) were selected for immunohistochemistry.

With the purpose of measuring morphological parameters of microglial cells along with their IL-1β expression level, double immunofluorescence with IBA1 and IL-1β antibodies was performed. Free floating vibratome sections were washed with PBS, and non-specific binding sites were saturated with PBT solution (0.3% bovine serum albumin, 0.3% Triton X-100 in PBS pH 7.3). Primary antibodies (anti-IBA1, host: rabbit, WAKO, 19-19741 and anti-IL-1β, host: goat, R&D Systems, AF501NA) were co-incubated overnight at 4°C. Sections were washed with PBS and then incubated for 1.5 h with the secondary antibodies (anti-rabbit Alexa 488, host: donkey, Molecular Probes, A-21206; and anti-goat Alexa 594, host: donkey, Invitrogen, A-11058). Sections were washed with PBS, mounted onto gelatine-coated slides, cover slipped with the anti-fading agent Mowiol 4-88 (Calbiochem/EMD Chemicals) and stored at 4°C. Negative controls for the immunohistochemistry consisted in omitting the primary antibodies.

### Image Acquisition and Processing

Images of activated microglia from immunolabeled sections including the third ventricle were acquired using the inverted microscope LEICA SP5 II equipped with a confocal scan unit. Images were captured with a 63x oil immersion objective, using the following acquisition parameters: for the fluorochrome Alexa 488 (Iba1-green): Argon laser intensity 55%; Gain 641; Offset 0; and Detector PMT aperture: 500–550 nm. For the fluorochrome Alexa 594 (IL1β-red): Helium-Neon laser intensity 67%; Gain 1009; Offset 0; and Detector PMT aperture: PMT 605–656 nm.

Images were taken using the z-stack tool. The distance between planes was established after prior trials, aimed to get cellular profiles of sufficient resolution for the subsequent morphological analysis; such distance was set in 1 μm. For each microscopic field selected, a z-stack was obtained from 20 to 30 planes (1 μm apart) taken along the *z* axis.

**FIGURE 1:**
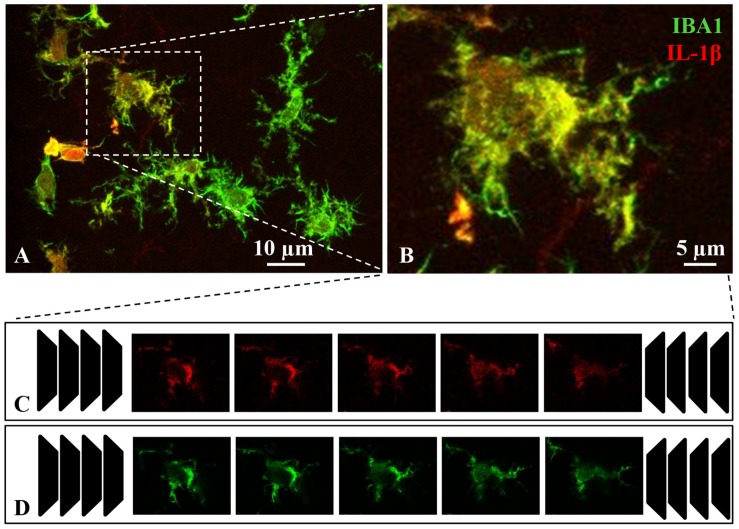
Procedure for the quantification of IL-1β staining in microglial cells. Brain sections containing the hypothalamus were immunostained with IBA1 (green channel) and IL-1β (red channel) antibodies. Activated microglial cells were evidenced by co-staining with both antibodies **(A)**. Activated microglia were randomly picked for image analysis **(B)**. Confocal z-stack images were captured, each containing 20–30 confocal planes **(C,D)**. After splitting the red and green channels, IL-1β intensity for an individual microglial cell was estimated on the red channel images **(C)**. Briefly, the intensity of all red-positive pixels in every plane was measured, and then the counted values of all planes were added up to obtain the total intensity staining per cell. Secondly, the counterpart green channel images **(D)** were used to calculate the cellular volume: the green-positive area was measured for each confocal plane (μm^2^), and then the cell volume was calculated knowing the distance between planes (≈ 1 μm). Finally, the ratio red staining intensity to cellular volume yielded the IL-1β expression level of a particular microglial cell, relative to its volume (intensity/μm^3^).

**FIGURE 2:**
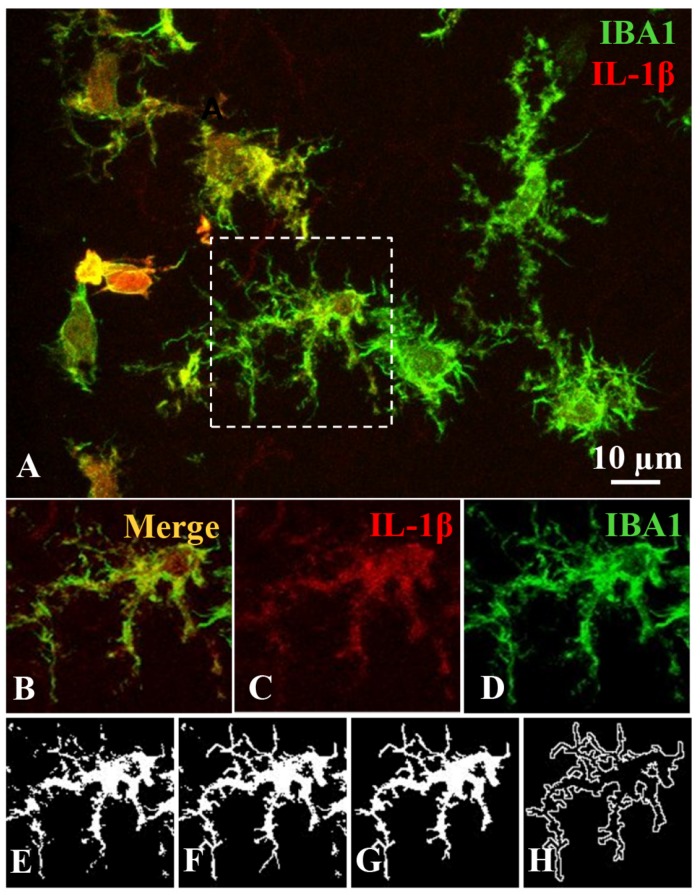
Image processing for an accurate morphometric analysis of individual microglial cells. This procedure was carried out on the same images used for quantifying IL-1β expression level **(A)**. For each single microglial cell, the z-stack planes were projected and grouped, and the resulting image **(B)** was color-splitted to get the cellular profile displayed by IBA1 staining (green channel; **D**) out of IL-1β label (red channel; **C**). The green-channel image **(D)** was transformed to a binary image **(E)** and then edited **(F)** to obtain a single field or area of pixels representing the filled **(G)** and the outlined **(H)** shapes of the cell. These filled and outlined figures were used to measure the morphometric parameters.

From these images, individual microglial cells were selected and cropped according to the following criteria: (i) random selection from the dorsal wall of the third ventricle, starting in the subependyma and moving toward the brain parenchyma up to a depth of about 100 μm; (ii) different intensities in the IL-1β label (as the study was focused on activated microglia, cells with no IL-1β label at all were excluded); (iii) no overlapping with neighboring cells; and (iv) complete nucleus and branches. The images of selected single microglial cells were processed in two different ways. On one hand, the intensity of the IL-1β label was quantified (see Section Quantification of IL-1β Label in Single Microglial Cells; Figure 1), and on the other hand an extensive morphological analysis of the same cell was performed (see Section Quantification of Morphological Parameters in Single Microglial Cells; Figure 2). For each type of analysis, a series of steps were performed using FIJI free software (freely downloadable from http://fiji.sc/Fiji).

### Quantification of IL-1β Label in Single Microglial Cells

To obtain an accurate measure of the IL-1β label in individual cells, the z-stack images (20–30 planes) from a selected cell (Figure 1B) were split into the red (Figure 1C, corresponding to IL-1 β) and the green (Figure 1D, corresponding to IBA1) channels. First, the intensity of IL-1β label was calculated by an arbitrary method. The *Raw Integrated Density* was measured (the sum of the values of intensity of the pixels in all the planes making up the whole cell). Secondly, the counterpart green channel images (Figure 1D) were used to estimate the volume of the cell as follows: to obtain the total positive area in μm^2^, the number of green-positive pixels of all planes in the z-stack was counted. Knowing that the distance between the z-planes was 1 μm, the volume of the cell was then calculated (in μm^3^). Finally, the IL-1β relative intensity of each cell was calculated by dividing its *Raw Integrated Density* between its volume. This approach intended to avoid bias due to changes of cell size occurring during the activation process.

### Quantification of Morphological Parameters in Single Microglial Cells

Each studied microglial cell was also processed for an accurate morphometric analysis. In this case, two-dimensional projection images were obtained from confocal z-stacks (Figure 2A). First, the double-color image (Figure 2B) was split to obtain the IBA1 label in the green channel, which quite accurately mirrors the cell profile (Figure 2D). This green-channel image was transformed into a binary image (Figure 2E), which was then manually edited (Figure 2F) to obtain a filled profile (Figure 2G) and an outlined shape (Figure 2H) of the cell (for more details see Fernández-Arjona et al., 2017). These filled and outlined figures were used to measure the morphologic parameters. For this purpose, the free software plugin FracLac for ImageJ was used (Karperien A., FracLac for ImageJ^1^. 1999–2013; available at the ImageJ website, National Institutes of Health; Karperien et al., 2013). The 15 parameters measured were: *fractal dimension*, *lacunarity*, *cell area*, *cell perimeter*, *cell circularity*, *convex hull area*, *density*, *convex hull perimeter*, *roughness*, *convex hull span ratio*, *convex hull circularity*, *bounding circle diameter*, *maximum span across the convex hull*, *the ratio convex hull radii* and *the mean radius*. All of them are explained in Supplementary Material, as well as a step guide for their measurement (Supplementary Figure S1 and Supplementary Material Methods. Fractal analysis; Fernández-Arjona et al., 2017).

### Statistical Analysis

#### Simple Linear Regression Analysis

To evaluate the correlation between each morphometric parameter and IL-1β label intensity in activated microglial cells, a simple linear regression analysis was performed. Morphological parameters were considered the dependent variables and the relative intensity of IL-1β was the independent one. The relationship between the independent variable and each dependent variable (15 different morphological parameters) was analyzed by pair-wise datasets of each cell, conducting an independent simple linear regression for each parameter. Regression coefficients [*F*_0__.__05__(__2__),__150__,__150_] of each function pointed out the dependence of the dependent variable (morphometric parameters) on the independent variable (IL-1β intensity). Dependence was considered significant when *P* < 0.05. Correlation coefficients [*r*_0__.__05__(__2__),__151_] measure the strength of the linear relationship between the two variables.

#### Hierarchical Cluster Analysis

With the aim of identifying likeness among NA-activated microglial cells and thus obtaining microglial subtypes, a hierarchical cluster analysis (HCA) was performed using the morphometric parameters previously measured. For this purpose, SPSS Statistics software, version 21.0 (Armonk, NY, United States; IBM Corp.) was used. Similarity appraises were generated by measuring the Euclidean distance (the square root of the sum of the squared differences between values for the items) following the Ward’s method (Ward, 1963) for interval data. All data were normalized in order to obtain values in a similar scale. A dendrogram plot based on the Euclidean distance was used to display the number of clusters proposed. To find out the more suitable parameters for separating our population of cells into different groups, the *multimodality index* (*MMI*) of each parameter was calculated (Scheweitzer and Renehan, 1997) using the following formula:

MMI=[M3+21]/[M4+3(n-1)/2(n-2)(n-3)]

where n is the sample size (in this case 250 cells), M3 is *asymmetry* (or *skewness*; *A*) and M4 is *kurtosis* (*K*), obtained from dispersion data analysis. The number of appropriate clusters was estimated by the Thorndike procedure (Thorndike, 1953). Briefly, the average within-cluster distance is plotted for different numbers of clusters, resulting in a curve that shows a decrease in the distance as the number of clusters increases. The number of clusters finally selected is revealed by a sudden flattening of the curve in the plot.

#### Linear Discriminant Analysis

Linear discriminant analysis (LDA) is a statistical method used for pattern recognition, to characterize or separate two or more groups, and also to create a function able to distinguish, as accurately as possible, the pertaining to each group. The evaluation by LDA of 250 NA-activated microglial cells was carried out by SPSS Statistics software. The following equation shows the linear discriminant functions:

LD=A1X1+A2X2+…⁢AnXn+C

where An is a coefficient of each individual morphometric parameter, Xn is each variable (the morphometric parameter value) and C is a constant. The discriminant functions were considered satisfactory when the predictive ability to discriminate exceeded 80%. Wilk’s lambda was used to test for significant differences between the groups for each individual predictor variable. The values of standardized coefficients show the net contribution of each variable to the discriminant function. The centroid of each group and the boundaries in the territorial map describes the predicted groups (Fisher, 1936; Yamada and Jinno, 2013; Ohgomori et al., 2016).

#### Analysis of Variance

Comparisons of data means were carried out using SPSS Statistics software. The Kolmogorov–Smirnov normality test, along with the Levene homoscedasticity test, was used to verify that data could be analyzed by parametric methods. One-way analysis of variance (ANOVA) was used to compare the morphometric parameters means of the different clusters. Pair-wise multiple comparisons were performed by Bonferroni test. Differences were considered statistically significant when the *P* obtained was <0.05.

## Results

### Diverse Expression Levels of the Pro-inflammatory Cytokine IL-1β Are Observed in Hypothalamic Activated Microglial Cells

**FIGURE 3:**
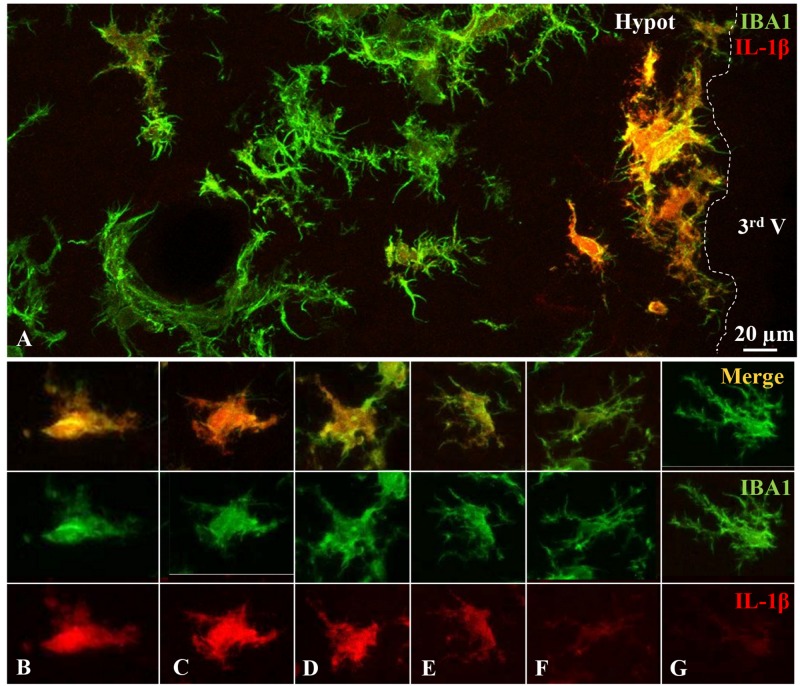
Varied expression level of the inflammatory cytokine IL-1β by hypothalamic microglial cells. Double immunofluorescence staining on brain sections obtained from rats ICV-injected with NA. Twelve hours after NA-injection, a broad co-localization of IL-1β (red) in IBA-1 positive (green) hypothalamic cells can be observed **(A)**. In sub-ependymal and periventricular areas, microglial cells similarly stained with IBA1 displayed diverse IL-1β staining intensities, which ranged from high **(B,C)**, or medium **(D,E)** to low **(F,G)**. Hypot, hypothalamus; 3^rd^ V, third ventricle.

The ICV administration of NA results in the activation of microglial cells located nearby the ventricular walls, activation that is evidenced by IL-1β expression. Double immunofluorescence showed a broad co-localization of IL-1β in IBA1 positive cells (which mostly correspond to microglia) located in periventricular areas, particularly of the hypothalamus (Figure 3). Control animals injected with saline did not show any IL-1β staining (results not shown). Since NA was injected within the right lateral ventricle and then distributed to other brain areas by the rostro-caudal cerebrospinal fluid flow, the IL-1β positive microglia could be observed in the hypothalamus parenchyma close to the third ventricle. Cytokine positive microglial cells were found up to about 100 μm under the ventricular surface, but not further deep, confirming that non-activated cells were also present in this model. The IL-1β and IBA1 positive cells most probably correspond to microglial cells, rather than infiltrated cells, because (1) 12 h after ICV injection infiltration occurs at the lateral ventricle, but not in the hypothalamus, (2) these cells are extensively ramified, and it is unlikely that a peripheral cell might infiltrate in the lateral ventricle, flow to the hypothalamus, migrate through the ependymal layer, and ramify in just 12 h. On the other hand, IL-1β/IBA1 positive cells remarkably presented variable degrees of IL-1β expression, ranging from quite high (Figures 3B,C) and medium (Figures 3D,E) to almost none (Figures 3F,G). With a naked eye, microglial cells with lower IL-1β expression bore more and longer ramifications (Figures 3F,G). Cells with higher IL-1β expression were usually located closer to the ventricle. However, an objective method to assess the activation of microglial cells would be more appropriate. Therefore, we aimed to correlate quantitative morphometric parameters with IL-1β expression level. To do so, microglial cells double-labeled with IBA1 (Figure 3, green) and IL-1β (Figure 3, red) were randomly picked from the periventricular region of the hypothalamus of rats injected with NA 12 h before.

### Morphological Parameters Correlate With the Level of Expression of IL-1β in Hypothalamic Microglia From NA-Injected Rats

We have previously verified significant differences in morphological parameters upon transition of microglial cells to an NA-induced activation state (Fernández-Arjona et al., 2017). Here we pursued to investigate if the broad range of values of the different morphological parameters might correlate to different degrees of IL-1β expression. That is, if the gradual morphological change could be associated to a progressive increase of microglial activation, evidenced by the expression of IL-1β. To demonstrate this, fifteen morphological parameters were analyzed in individual microglial cells sampled from the hypothalamus. In those same cells, the level of expression of IL-1β was also measured (IL-1β label, measured as *Raw Integrated Density*, relative to the total cell volume, the later calculated by means of IBA1 labeling). The regression analysis illustrates how the value of a dependent variable varies when the value of an independent variable changes. In this study, the independent variable was the level of IL-1β expression, and the morphological parameters were the dependent variables. Thus, for each morphological parameter, the values of the parameter over the IL-1β relative intensity were analyzed by a simple linear regression of the paired microglial dataset.

**FIGURE 4:**
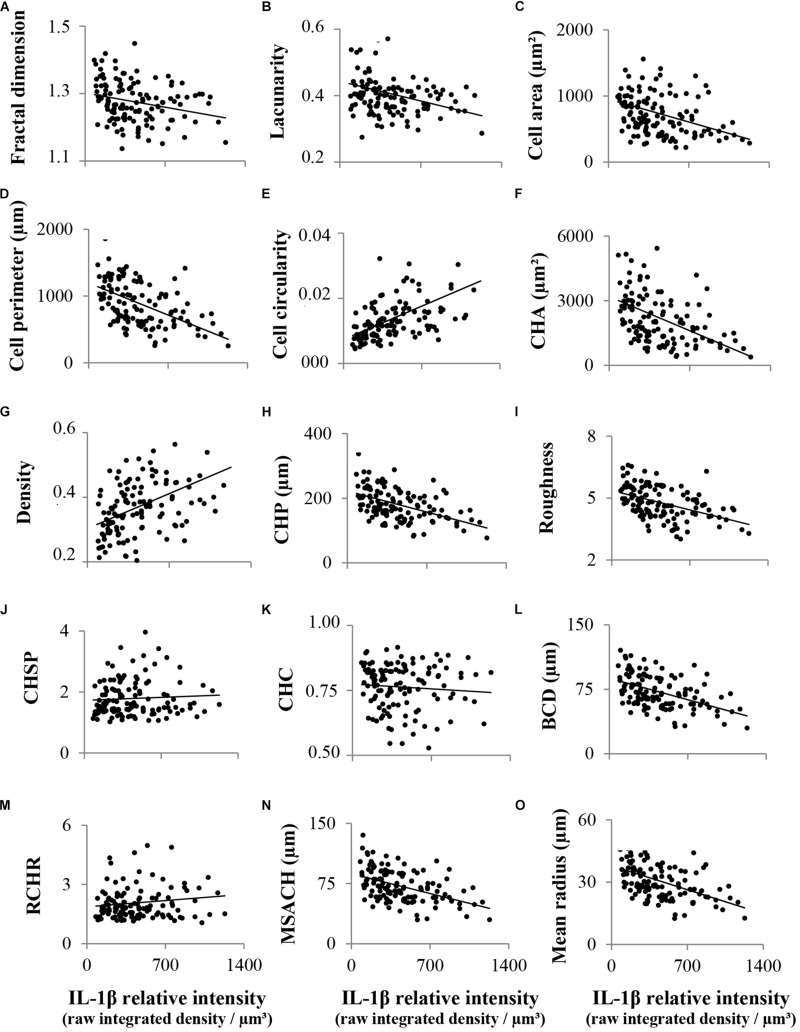
Linear regression analysis between different microglial morphological parameters and IL-1β staining intensity. Fifteen morphological parameters as well as IL-1β relative staining intensity were measured in a total of 150 activated hypothalamic microglial cells. These cells were plotted in scatter graphs, where each morphological parameter was the dependent variable and IL-1β staining intensity (measured as the *Raw Integrated Density* relative to cellular volume) was the independent variable. The parameters *fractal dimension* **(A)**, *lacunarity* **(B)**, *cell area* **(C)**, *cell perimeter* **(D)**, *convex hull area* (*CHA*; **F**), *convex hull perimeter* (*CHP*; **H**), *roughness* **(I)**, *bounding circle diameter* (*BCD*; **L**), *maximum span across the convex hull* (*MSACH*; **N**), and the *mean radius* **(O)**, showed a negative dependence on IL-1β expression, while the parameters *cell circularity* **(E)** and *density* **(G)** had a positive dependence on the same independent variable. Regression analyses were significant for all these parameters, and correlation coefficients were significantly strong for the straight-line relationship between each paired variables. On the contrary, there was no dependence for the *ratio convex hull radii* (*RCHR*; **M**), the *convex hull circularity* (*CHC*, **K**), and the *convex hull span ratio* (*CHSR*; **J**).

Hence, the functional dependence of each variable on IL-1β expression was tested by analysis of variance computing the sum of squares by a linear regression, for a population of 150 microglial cells (Figure 4 and Table 1). The parameters *fractal dimension* (Figure 4A), *lacunarity* (Figure 4B), *cell area* (Figure 4C), *cell perimeter* (Figure 4D), *convex hull area* (*CHA*, Figure 4F), *convex hull perimeter* (*CHP*, Figure 4H), *roughness* (Figure 4I), *bounding circle diameter* (*BCD*, Figure 4L), *max span across the convex hull* (*MSACH*, Figure 4N) and *the mean radius* (Figure 4O) showed a negative dependence on IL-1β relative intensity, while the parameter *cell circularity* (Figure 4E) and *density* (Figure 4G) had a positive dependence on the same independent variable; regression analyses were significant for all these parameters (Table 1). However, there was no dependency for the *convex hull span ratio* (*CHSR*, Figure 4J), the *convex hull circularity* (*CHC*, Figure 4K) and the *ratio convex hull radii* (*RCHR*, Figure 4M) versus IL-1β relative intensity. Regression coefficients (*F*) of those functions pointed out a significant dependence of 12 morphometric parameters on IL-1β expression (Table 1); three parameters (*the ratio convex hull radii*, *convex hull circularity* and *convex hull span ratio*) did not show any dependence. Correlation coefficients (*r*) were used to calculate the coefficients of determination (*r*^2^) which indicate the strength of the linear relationship between the two variables, and therefore the reliability of the prediction of the dependent variable based on the independent variable. The results showed significant correlations for each linear regression (Table 1) except for the three parameters above mentioned (*the ratio convex hull radii*, *convex hull circularity* and *convex hull span ratio*). Regression analysis is widely used to make predictions. Given that most of the morphological parameters used here present a statistically significant dependence on IL-1β relative intensity, it could be inferred that microglial morphological changes are closely related to the extent of the inflammatory activation of the cell. Also, for a population of microglial cells, these parameters might be considered as good predictors of the expression level of this cytokine. However, such prediction would not be reliable in the case of single microglia because, as can be observed in the plots (Figure 4), a relatively high dispersion of data exists.

**TABLE 1:** Statistics of simple linear regression analyses performed between different morphological parameters and IL-1β relative intensity of a population of activated microglial cells.

**Parameter**	***F***	***P* significance**	**Regression line**	***r*^2^**
Cell perimeter	40.3	3.7 × 10^–9^	*Y* = −0.68*X* + 1195	0.245^∗∗∗^
Cell circularity	37.9	9.5 × 10^–9^	*Y* = −1 × 10^–5^*X* + 0.007	0.234^∗∗∗^
*Mean radius*	37.1	1.3 × 10^–8^	*Y* = −0.017*X* + 3	0.231^∗∗∗^
Convex hull perimeter	36.1	1.9 × 10^–8^	*Y* = −0.093*X* + 222	0.226^∗∗∗^
Bounding circle diameter	34.7	3.5 × 10^–8^	*Y* = −0.036*X* + 88	0.212^∗∗∗^
Max span convex hull	32.5	8.4 × 10^–8^	*Y* = −0.035*X* + 87	0.209^∗∗∗^
Roughness	32.2	9.3 × 10^–8^	*Y* = −1.3 × 10^–3^*X* + 5.4	0.206^∗∗∗^
Convex hull area	29.3	3.1 × 10^–7^	*Y* = −2.26*X* – 3186	0.191^∗∗∗^
Density	26.7	9.2 × 10^–7^	*Y* = −2 × 10^–4^*X* + 0.3	0.177^∗∗∗^
Cell area	18.9	2.9 × 10^–5^	*Y* = −0.48*X* + 953	0.132^∗∗∗^
Lacunarity	14.3	2.3 × 10^–3^	*Y* = 8 × 10^–5^*X* + 0.44	0.104^∗∗∗^
Fractal dimension	9.1	0.003	*Y* = 6 × 10^–5^*X*+	0.068^∗∗^
The ratio convex hull radii	1.7	0.19	*Y* = 4 × 10^–4^*X* + 1.87	0.013
Convex hull circularity	0.7	0.39	*Y* = −3 × 10^–5^*X* + 0.77	0.006
Convex hull span ratio	0.5	0.49	*Y* = −1 × 10^–4^*X* + 1.73	0.004

*Regression dependence by *F-test* procedure was significant when *P* < 0.05. Coefficient of determination (*r*^2^) was used as a measure of the strength of the straight-linear relationship (^∗∗∗^*P* < 0.001; ^∗∗^*P* < 0.005).*

*Fractal dimension* (*D*) was determined by the box counting method. Previous studies revealed that small changes in *D* values are related to subtle changes in the complexity of branches (Karperien et al., 2013). In our study *D* data ranged from 1.15 to 1.40. High values of *D* corresponded with low cytokine expression. The correlation analysis showed a tendency of *D* to decrease as IL-1β expression increases. This suggests that microglial cells with low NA-activated state (low IL-1β) present phenotypes associated to high values of *D*, like de-ramified, hypertrophied or bushy. When activation escalates (lower values of *D*), microglia profile gradually modifies toward un-ramified. In addition, in our sample the higher value of *D* obtained was 1.45, indicating that highly branched profiles, with small soma and quite fine and long processes, are rare amongst NA-activated hypothalamic microglial cells.

*Lacunarity* (Λ) assess the heterogeneity of the cell profile. Low Λ values reflect homogeneity and high Λ measures mean heterogeneity (Karperien et al., 2011). In our sample, microglial cells with lower expression of IL-1β presented higher Λ values. Also, the regression analysis pointed out a decrease in Λ in parallel to IL-1β intensity increases. Therefore, microglia with mild NA-activation state present a heterogeneous cellular profile (high Λ values), and as the level of activation increases their shape tends to be more homogeneous (low Λ values).

*Cell area* and *cell perimeter*, as well as the *area* and the *perimeter* of the *convex hull*, mainly describe the cell size. In the case of NA-activated microglial cells high values of those parameters have been found in cells with low IL-1β expression. The regression analysis showed a decrease in cells size in parallel with increases in the expression of the pro-inflammatory cytokine. These results indicate a progressive reduction of microglial size as the activation state of the cells enhances.

These cell-size changes were complemented with the morphological measures of *density* and *roughness*. On one side, low values of *density* were accompanied with low cytokine expression; both variables presented a positive correlation. Therefore, the more compact the profile of the cell, the more activated it is. On the other hand, the parameter *roughness* showed the opposite behavior: high *roughness* values relate to low IL-1β intensity, and thus the regression analysis pointed out a negative correlation between both variables. This denotes that microglial cell surface irregularities tend to decrease in parallel with the progressive enhancement of its activation.

These results confirm that, in microglial cells, a correlation exists between different morphological parameters and the degree of inflammatory activation. We next sought to explore the possibility that there are different microglial morphotypes related to various degrees of activation.

### Hierarchical Cluster Analysis Allows Identifying Four Types of Activated Microglial Cells Based on Morphological Parameters

The choice of suitable variables is critical for the outcome of HCA. For this reason we initially considered the fifteen parameters above mentioned, and examined their frequency distribution in a histogram (Supplementary Figure S2). Each probability distribution was compared with a normal distribution, and *asymmetry* (or *skewness*) together with *kurtosis* were calculated, both of which allowed to estimate the *multimodality index* (*MMI*) of each parameter (see section Materials and Methods). The *MMI* gives an idea of the distribution of the data around one or multiple values. Thus, parameters with *MMI* > 0.55 are multimodal and therefore suitable to perform cluster analysis (Scheweitzer and Renehan, 1997). Based on their *MMIs*, only the following three morphometric parameters were appropriate for the cluster analysis: *lacunarity*, *cell circularity* and *convex hull span ratio* (Table 2).

**TABLE 2:** Multimodality indexes of morphological parameters.

**Parameter**	**Multimodality index**
Lacunarity	0.587^∗^
Cell circularity	0.560^∗^
Convex hull span ratio	0.552^∗^
The ratio convex hull radii	0.522
Convex hull area	0.503
Cell area	0.497
Convex hull circularity	0.456
Density	0.438
Cell perimeter	0.431
Max span convex hull	0.417
Bounding circle diameter	0.414
Mean radius	0.410
Convex hull perimeter	0.402
Fractal dimension	0.385
Roughness	0.372

*^∗^Parameters with a *MMI* > 0.55 presented a multimodal distribution.*

**FIGURE 5:**
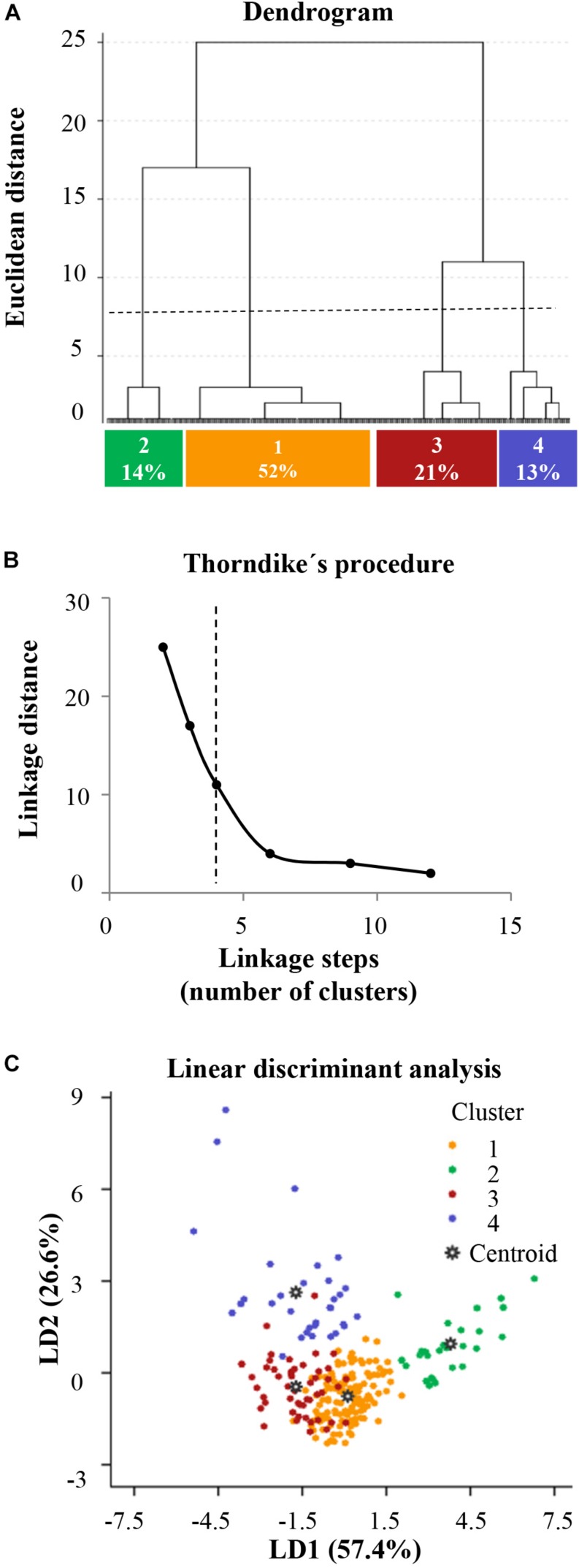
Activated microglia can be classified according to morphological parameters. A hierarchical cluster analysis (HCA) was performed on 250 NA-activated microglial cells sampled from the rat dorsal hypothalamus. This dissimilarity measure was based on three suitable morphological parameters selected according to their multimodality index (*MMI*) value. The clustering results are presented in a dendrogram **(A)**, where the abscissa represents individual microglial cells and the ordinate corresponds to the linkage distance measured as Euclidean distance. The horizontal dashed line denotes the cut off for four clusters, numbered 1 through 4, which are color coded as orange, green, dark red and blue, respectively. The percentage indicates the frequency distribution of microglial cells in these clusters. **(B)** A plot of linkage distance vs. linkage steps (or number of clusters) was performed following Thorndike’s procedure. The vertical dashed line points out a marked decline in the slope, which indicates that four is an appropriate number of clusters. **(C)** Territorial mapping of microglial cells on the plane explained by the first two linear discriminant functions (LD1 and LD2); the proportion of trace for each LD is shown in parenthesis. The cells are color coded based on their cluster allocation; the centroids represent the mean value of each cluster.

The HCA performed on z-transformed data sets of the three selected parameters yielded a dendrogram based on the Euclidean distance between groups, using the Ward’s method (Figure 5A). The Thorndike’s procedure (Thorndike, 1953) was applied to establish the appropriate number of clusters (Figure 5B). This method uses the representation of linkage distance versus linkage steps (or number of clusters); a sudden decrease in linkage distance occurs at a certain number of clusters, which is evidenced by a marked flattening of the curve. In our case, this happens when the number of linkage steps is four (dashed line in Figure 5B). Microglial cells were thus classified into four clusters.

Next, we searched for linear discriminant functions which could explain the variance, and that could also suggest the variables that are more relevant for discrimination, i.e., that have the highest predictive capacity. This search resulted in two functions (Table 3). The linear discriminant function 1 (LD1), with a correlation of 0.869, which explained 57.4% of variance, and the linear discriminant function 2 (LD2), with a correlation of 0.767, described 26.6% of variance. Thus, both functions together accumulated 84% of variance, remaining unexplained only 16% of it. Moreover, Wilks’s lambda and chi-squared pointed out significant differences between the means of the compared groups (Wilks’s lambda = 0.054; χ^2^ = 718.9; *df* = 9; *P* < 0.001). The discriminant functions include coefficients for each variable, which are listed in Table 3. The value of those coefficients indicates the partial contribution of each variable to the function, that is, the importance of each variable as predictor of cell sorting into the four clusters, with a higher absolute value indicating a better predictive variable. LDA revealed that *lacunarity* and *cell circularity* are the critical parameters when sorting microglial cells. The discriminant scores of 252 microglial cells were determined for LD1 and LD2, and plotted in a territorial map using a color code to identify each cluster (Figure 5C). This type of graph illustrates how cells within each cluster are grouped around a centroid (the cluster mean).

**TABLE 3:** Linear discriminant functions.

**Parameter**	**LD1**	**LD2**
Lacunarity	0.85^∗^	0.575
Convex hull span ratio	−0.408	–0.25
Cell circularity	−0.254	1.012^∗^
Proportion of trace (%)	57.4	26.6

*^∗^The highest absolute values indicate the parameters with greater influence.*

**FIGURE 6:**
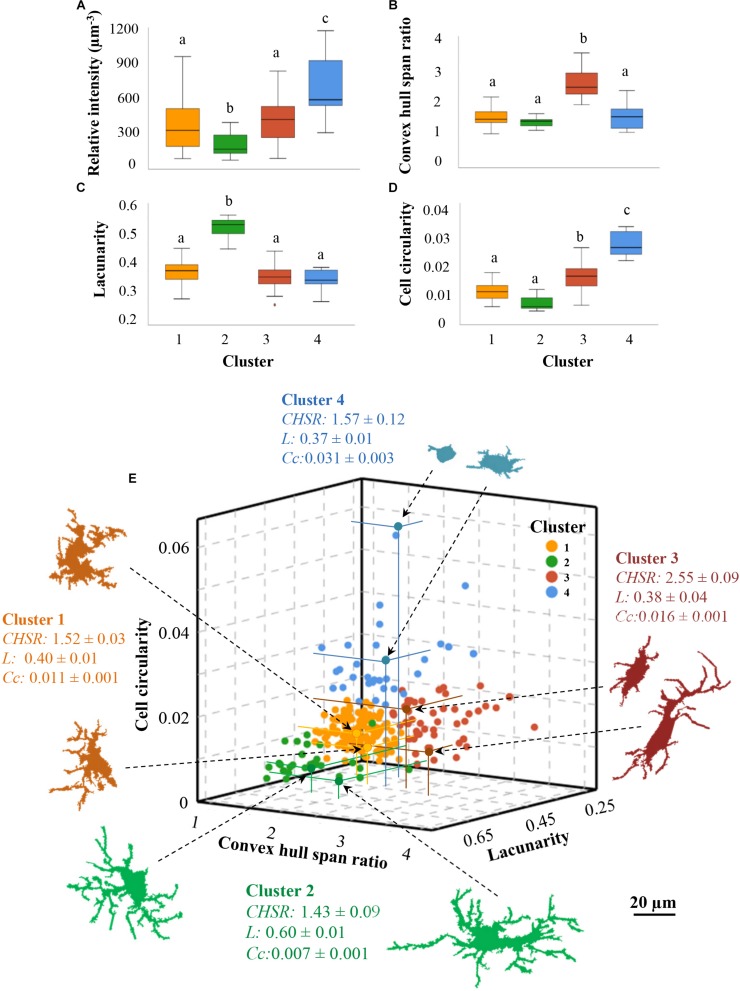
The microglial cells clusters defined by morphological parameters are also different regarding IL-1β expression. The IL-1β relative intensity previously calculated for each microglial cell was represented in a boxes and whiskers plot, showing the mean value (±SD) in each of the morphologically established clusters **(A)**; the average IL-1β relative intensity was lowest in microglia belonging to cluster 2, and highest in those of cluster 4. Mean values of the morphological parameters used for clustering were also different **(B–D)**. Letters on top of each box indicate pair comparisons results: means of boxes with different letters are statistically different (*P* < 0.05). **(E)** Data points of a total of 250 activated microglial cells were plotted as a function of the three multimodal variables: *cell circularity* (*Cc*), the *convex hull span ratio* (*CHSR*), and *lacunarity* (*L*), on the y, x and z axes, respectively. A fourth dimension was set by a color code as a function of microglia cluster allocation. This three-dimensional Cartesian coordinate reveals a specific territorial distribution of the cells belonging to each cluster. Exemplary color-coded microglia profiles from each cluster are presented; the arrows point the location of those particular cells in the graph, and the distance to each axis is highlighted with colored lines; next to them, the cluster mean values ± standard deviation of each parameter are also shown.

Once HCA and LDA had yielded four groups of hypothalamic activated microglial cells, we tested if they were actually different morphotypes, comparing their parameters means using one-way ANOVA. The comparison of the four clusters regarding IL-1β relative intensity (Figure 6A) showed in an objective manner that they were different in terms of their activation status. Therefore, each cluster or morphotype could be assigned to a particular level of inflammatory activation. Although clusters 1 and 3 were not different regarding IL-1β expression, they were different from clusters 2 and 4, and were also different regarding morphological parameters (*convex hull span ratio* and *cell circularity*; Figures 6B,D). Consequently, each morphotype could be assigned a particular level of activation as follows: cluster 2 represents a low activation state, clusters 1 and 3, although morphologically different, fit with intermediate activation states, and cluster 4 appertain to the highest activation level.

Comparisons of each cluster means were also done for all the morphological parameters measured (Supplementary Figure S3). One-way ANOVA showed significant differences between clusters in most parameters, what supports that each cluster represents a particular morphotype. To simplify, only the discriminating parameters used to perform HCA and LDA were presented (Figures 6B–D). The cells in cluster 1 do not present any trait that allows distinguishing them from the rest of the cells groups, since their morphometric values are intermediate in comparison to the remaining groups; this is in accordance with their intermediate level of activation, as previously exposed. Cells in cluster 3, although also described as mild activated cells (similarly to cluster 1), showed however an increased *convex hull span ratio* that distinguished them from the remaining groups (Figure 6B). The main characteristic of cells in cluster 2 was the high *lacunarity* (Figure 6C), while cluster 4 cells exhibited the highest values of *cell circularity* (Figure 6D).

Therefore, different morphotypes were associated to each cluster, each of them described by different values of the most discriminant morphometric parameters (Figure 6E). According to those parameters, (a) microglial cells in cluster 3 are quite elongated or even rod shaped (red cells in Figure 6E); (b) microglial cells in cluster 2 present a heterogeneous or polarized morphology (green cells on Figure 6E); (c) cells belonging to cluster 4 are mostly rounded or amoeboid (blue cells on Figure 6E); (d) while those included in cluster 1 do not present a characteristic trait appearing quite similar to those in cluster 2 except that they are not elongated (yellow cells on Figure 6E). Since each cluster has been previously assigned a certain level of inflammatory activation, a particular microglial cell morphotype (defined by morphometric values) could be assigned to each level of inflammatory activation.

## Discussion

In microglial cells, the expression of the cytokine IL-1β reveals its polarization toward a pro-inflammatory phenotype that appears within few hours of exposure to a specific stimulus (Olah et al., 2011; Walker et al., 2014; Orihuela et al., 2016). In our experimental model, the injection of NA into the lateral ventricle generates an acute inflammatory process that involves the activation of microglia, which is evidenced by IL-1β expression (Granados-Durán et al., 2015; Fernández-Arjona et al., 2017). In this study, fluorescence immunohistochemistry showed that microglial cells located nearby the ventricular surfaces specifically express this inflammatory cytokine 12 h after the administration of NA, while in deeper parenchymal areas the IL-1β label was practically absent. In the region selected for this study, the dorsal hypothalamus, activated microglia was found in the nervous parenchyma up to 100–200 μm from the ventricular surface. This fact suggests that the NA spreading from the ventricular cavities toward the nervous parenchyma is responsible for the activation of microglia.

With the aim of identifying features of activated microglial cells, some studies showed that activated cells exhibit morphological changes compared to the typical surveillant morphotype of resting microglia (Boche et al., 2013; Walker et al., 2014). Those studies were accomplished by either subjective (Kloss et al., 2001; Cho et al., 2006; Diz-Chaves et al., 2012; Rodríguez-Arias et al., 2018) or objective (Karperien et al., 2013; York et al., 2018) approaches, the latter employing quantitative morphological analysis. However, the existence of different cellular morphotypes within the population of activated microglial cells has not been previously addressed. In our NA-induced inflammation model it was evident that the IL-1β positive microglial population was heterogeneous both morphologically and regarding cytokine expression. Therefore, we aimed to analyze whether different morphotypes could be defined within the activated microglial population and, moreover, if they could be assigned to different levels of activation. With a naked eye, most of the IL-1β-positive microglial cells selected for this study showed a hypertrophied soma with variety of branching patterns and, apart from rare exceptions, all were far from round or amoeboid. These observations suggested that all microglial cells selected for this analysis were in an inflammatory state of activation, but exhibiting different degrees of activation and a wide range of morphologies.

Traditionally, two forms of microglia have been described: one considered as a resting state in which cells have a highly branched morphology, and another considered as an activated state in which they acquire an amoeboid form (Davis et al., 1994; Sierra et al., 2016). Other studies have described activated microglia as cells with hypertrophied (Ayoub and Salm, 2003) or even cylindrical shape (Wierzba-Bobrowicz et al., 2002; Taylor et al., 2014). Under normal conditions, microglia may display various functional states (Ransohoff and Perry, 2009; Gomez-Nicola and Perry, 2015). Currently, it is widely accepted that highly branched microglia are not in a resting state as initially thought, but are greatly dynamic in their role of monitoring the environment (Nimmerjahn et al., 2005; Nimmerjahn, 2012). This scenario is even more intricate, since it has been observed that in some pathological situations microglial cells show an overall reduction of their processes length (Madore et al., 2013), although in others they preserve the ramifications (Stence et al., 2001; Petersen and Dailey, 2004). On the other hand, it has been observed that, after a traumatic brain injury, microglial prolongations converge toward the site of the lesion without occurring a displacement of the cell body, thus establishing a potential barrier between the healthy and the injured tissues (Davalos et al., 2005). Therefore, microglial cells with intermediate morphotypes, such as those with abundant ramifications in situations of brain damage, have been consistently described. These intermediate forms of activated microglia seem to be also the predominant morphotypes in our acute inflammation model. At first sight, the population of hypothalamic microglia activated by NA seems to be branched morphs of the hypertrophied and shrub type. Conversely, there are no surveillant ramified nor hyper-ramified (a morphology found in chronic stress; Hinwood et al., 2013) microglial cells. Likewise, hypothalamic amoeboid microglia are virtually absent in our model.

### Morphological Parameters Correlate With the Level of Expression of IL-1β in NA-Activated Hypothalamic Microglia

Fifteen morphometric parameters were measured in single cells sampled from the dorsal hypothalamus, along with the quantification of the level of expression of IL-1β by an image processing method designed to account for minor differences in the immunostaining intensity. To investigate if there was a gradual morphological change that paralleled the activation level in microglial cells, a simple linear regression analysis was performed to search for a correlation between each individual morphometric parameter and the level of expression of IL-1β (as an indicator of pro-inflammatory activation). The regression analysis results showed that, for most morphometric parameters (dependent variable), there is a dependence on the relative intensity of IL-1β (independent variable). That is, upon activation, the increase in the level of expression of IL-1β may predict how the morphology of a population of microglial cells progressively changes. Thus, it seems more accurate to think of a continuum of gradual morphological changes coming about with a gradual increase in activation, more than simply referring to an activated state. The results from this analysis demonstrate, in an objective manner, a long assumed statement: that microglial cells change their shape when they become activated. However, our results support such correlation for a population of microglial cells. Therefore, while morphological parameters could predict the level of activation of a population of cells, such prediction would not be reliable in the case of individual microglial cells, because of the relatively high dispersion of data (paramerters’ values) amongst microglia.

The meaning of the gradual changes of individual morphological parameters or, even more, several parameters simultaneously changing, is not easy to unravel. However, in an attempt to visualize and understand the morphological changes, the parameters may be related to the different features of cell’s shape. Thus, the variables that significantly change with the level of microglial activation are related to the regularity of the cell’s surface (*cell perimeter* and *roughness*), the complexity of ramifications (*fractal dimension*), the heterogeneity of the shape (*lacunarity*), the soma thickness (*cell circularity* and *density*) and the cell’s size (*mean radius, convex hull perimeter, bounding circle diameter, max span convex hull, convex hull area*, and *cell area*). No correlation was observed in the parameters related to the cylindrical shape of the cells (*convex hull span ratio* and *the ratio of convex hull radii*), hence this feature is independent of the inflammatory activation degree of microglial cells. Our correlation results indicate, in an objective way, that when microglial cells become increasingly activated the complexity of their ramifications and their heterogeneity decrease, and they become more compact. However, it should be emphasized that the correlation analysis does not allow identifying different morphotypes, nor allows considering several morphological parameters simultaneously. These objectives, which are not the aim of correlation analysis, were therefore approached by HCA.

### Hierarchical Cluster Analysis Allows the Identification of Four Types of Activated Microglial Cells Based on Morphological Parameters

After showing microglial morphology dependence on the inflammatory activation level, we proceeded to seek for subgroups of microglial cells sharing similar traits within the population of activated microglia. Among all the morphological variables used in the previous correlation analysis, only *lacunarity*, *cell circularity* and *convex hull span ratio* were suitable to discriminate cellular subtypes. Hence, by using these parameters through HCA and LDA, four different clusters of NA-activated microglia were objectively revealed (Figure 5). Each cluster was further identified by particular average values of those three morphological parameters, as well as by a specific value of IL-1β relative intensity (that is, level of activation). Therefore, each cluster may be considered as a particular microglia morphotype within the activated hypothalamic population (Figure 6). The classification of activated microglia is relevant because each subgroup could be related to a particular physiological role.

Among the morphological parameters measured here, several of them can be used to discriminate between the arisen clusters (Supplementary Figure S3). However, in order to define each group and make these results more comprehensible, we preferred to take into account the most discriminating parameters (*lacunarity*, *cell circularity* and *convex hull span ratio;* Figure 6). Consequently, a slightly activated microglial group (cluster 2) is characterized by the greatest heterogeneity (*lacunarity*), and a highly activated microglial group (cluster 4) can be distinguished by the highest *cell circularity*. A third group includes cells with intermediate activation level (cluster 1 and 3), which can be subdivided in two other groups according to morphology (particularly the *convex hull span ratio*): cluster 3 includes rod-shaped cells, and cluster 1 less elongated cells.

Therefore, four groups of microglial cells were defined by their inflammatory activation level and by specific morphological parameters, namely *lacunarity*, *cell circularity* and *convex hull span ratio*. *Lacunarity* indicates the homogeneity (low values) or heterogeneity (high values) of an object (Karperien et al., 2011). If that object happens to be a cell, *lacunarity* defines the polarization of the cell, that is, when their prolongations are oriented toward a specific point. In our sample of hypothalamic activated microglia the morphotype with the highest *lacunarity* shows the lowest activation (cluster 2). This morphotype was also distinguished by the highest values of *fractal dimension* (Supplementary Figure S3A) which denotes a high level of complexity in ramifications (Karperien et al., 2013). The analysis of branch complexity has been used to discriminate whether microglial cells are resting or activated. Some researches employed this useful tool in particular situations, such as aged brains (van Olst et al., 2018), ischemic processes (Morrison and Filosa, 2013; Heindl et al., 2018), Alzheimer’s models (Baron et al., 2014) or acute pro-inflammatory processes (Verdonk et al., 2016). In those studies ramifications complexity (*fractal dimension*) decrease when microglial cells are activated. In the present study the higher branches intricacy and cell polarization coincides with slightly activated microglia (cluster 2) and, conversely, the morphotypes with intermediate and high activation (clusters 1, 3, and 4) match with decreased processes arborization. Hence, the assessment of branches complexity by *lacunarity* and *fractal dimension* contributes to describe the different morphotypes of activated microglia.

*Cell circularity* or *roundness* assesses whether microglial cells resemble an amoeboid form, where values closer to 1 indicate a more circular morphology. It is known that microglial dynamic conversion from resting to activated is accompanied by a retraction of the cellular processes into the cell body (Kettenmann et al., 2011). The subjective (Davis et al., 1994; Kloss et al., 2001; Norden et al., 2016) and objective (Kozlowski and Weimer, 2012; Davis et al., 2017; Fang et al., 2018) evaluation of the circularity have shown that *roundness* is directly related to microglial activation. Similarly, in the present study the greater the circularity, the higher the degree of inflammatory activation, a fact that is clearly evidenced in cluster 4.

The *convex hull span ratio* (*CHSR*) is the ratio between the longest and the shortest diameters of the convex hull containing the microglial cell. Therefore, it reflects the degree of elongation (and conversely, circularity) of the cell. This parameter was useful to discriminate a subgroup of microglia known as bipolar or rod microglia, which has a sausage-shaped soma with long thin processes (Taylor et al., 2014). Here, a morphotype of rod-microglial cells (cluster 3) was defined by the highest *CHSR* compared to the other clusters. However, it was indistinguishable from cluster 1 in terms of activation (i.e., both showed intermediate activation based on IL-1β expression). Hence, according to this result, acquiring a more or less pronounced cylindrical profile is not related to the activation level of microglial cells. The physiological function of rod microglia seems to be enigmatic. Plenty rod microglia have been observed in human brains affected by neurological diseases (Wierzba-Bobrowicz et al., 2002), and their abundance has been associated with aging (Bachstetter et al., 2017). Moreover, rod microglial cells arise after brain injury (Morrison et al., 2017), and are distributed in lines, coupled and adjacent to the cytoarchitecture of dendrites and axons (Ziebell et al., 2012). Interestingly, bipolar/rod-shaped microglia happen to be highly proliferative and, in the presence of LPS, quickly convert into amoeboid forms (within 30 min), suggesting that are crucial for repairing the damage (Tam and Ma, 2014). In our work, the elongated shape of cluster 3 microglia could be related to a sub-ependymal location rather than to the degree of activation, since they were always underneath the ependyma monolayer of the ventricular wall. Moreover, a conspicuous layer of sub-ependymal microglia, with relevant roles in periventricular insults, has been described (Carbonell et al., 2005), as well as the movement of IBA1 positive cells across the ependymal layer toward/from the ventricular cavities (Granados-Durán et al., 2015).

## Conclusion

This work demonstrates in a quantitative and objective way that the inflammatory activation of microglial cells is gradual, and that correlates with a morphological change. Even so, it is still possible to categorize activated cells according to their morphometric parameters, each category presenting a different activation degree. Whether the different activated microglial morphotypes undertake different physiological roles is a matter for future studies.

## Data Availability Statement

The raw data supporting the conclusions of this manuscript will be made available by the authors, without undue reservation, to any qualified researcher.

## Ethics Statement

Animal care and handling were performed according to the guidelines established by the Spanish legislation (RD 53/2013) and the European Union regulation (2010/63/EU). All procedures performed were approved by the ethics committee of the Universidad de Málaga (Comité Ético de Experimentación de la Universidad de Málaga; reference 2012-0013). All efforts were made to minimize the number of animals used and their suffering.

## Author Contributions

ML-Á, PF-L, and MF-A conceived and designed the study. MF-A and JG carried out the experiments, image acquisition, and image processing. ML-Á and MF-A analyzed the data and wrote the manuscript. All authors read and approved the final manuscript.

## Conflict of Interest

The authors declare that the research was conducted in the absence of any commercial or financial relationships that could be construed as a potential conflict of interest.

## Footnotes

1 http://rsb.info.nih.gov/ij/plugins/fraclac/FLHelp/Introduction.htm
