# Supplementary material for: Microglial Morphometric Parameters Correlate With the Expression Level of IL-1β, and Allow Identifying Different Activated Morphotypes
Source: Front Cell Neurosci. 2019 Oct 25;13:472. doi: 10.3389/fncel.2019.00472 (PMC6824358; doi:10.3389/fncel.2019.00472)
Supplement: Supplementary file 1 [file Data_Sheet_1.PDF]

Description of the morphological parameters

Microglial cells morphology was analyzed by FracLac for ImageJ plugin. This freely downloadable tool (see URL at the end of this section) serves to analyze 2-dimensional shapes in digital images and to measure morphological characteristics that are difficult to quantify. Regarding our work, a set of appropriate parameters can define the irregularity, elongation, roundness and circularity of individual microglial cells, as well as the complexity of its branches. The parameters analyzed in this work were:

1. ***Fractal dimension*** (*D*) is a recognized method to identify intermediate microglial forms ranging from simple-rounded to complex-branched (Karperien et al., 2013). A higher *D* means a greater complexity of the pattern. Box counting software was used to count the number of boxes containing any foreground pixels of the outlined pictures, which were processed along successively smaller calibre grids. The box size scale was obtained as power series; that is, the base is raised to the exponent added to it to make successive sizes. The slope finally obtained for each image was the average of twelve measurements with different and random placement of the grid.

2. ***Lacunarity*** (Λ) is associated with changes in the soma and additional morphological features. This parameter measures heterogeneity or translational and rotational invariance in a shape (Karperien & Jelinek, 2011). Low Λ value infers homogeneity, having the different parts of an image similar variance. On the contrary, high Λ measurements imply heterogeneity, containing the image many differently sized gaps or lacunas. The Λ calculated with the box counting software *FracLac* is a mass distribution of pixels from microglia images. The value of Λ obtained was a coefficient of variation expressed as pixel density per box as a function of box size. To avoid bias in Λ results, the mean of mass distribution by power series scales and also with twelve grid locations was calculated (Karperien & Jelinek, 2011).

3. ***Cell area*** was quantified as the total number of pixels present in the filled shape of the cell image, later transformed to squared micrometers (1 pixel area = 0.0132 μm^2).

4. ***Convex hull area***, where the convex hull is the smallest convex polygon (that with all interior angles smaller than 180°) containing the whole cell shape.

5. ***Density*** was calculated by dividing the *area* of the cell by its *convex hull area*. Some authors call this parameter *solidity*.

6. ***Cell perimeter*** is measured based on the single outline cell shape as the number of pixels expressed in microns (1 pixel side = 0.115 µm).

7. ***Convex hull perimeter*** is the single outline of the convex hull expressed in microns.

8. ***Roughness*** was calculated as the ratio of *cell perimeter* to *convex hull perimeter*.

9. ***Convex hull of span ratio*** is the ratio of the major to the minor axes of the convex hull. This parameter is also known as *form factor*.

10. ***Cell circularity*** was calculated as $(4\pi \times \textit{cell area}) / (\textit{cell perimeter})^2$. The circularity value of a circle is 1.

11. ***Convex hull circularity*** was similarly calculated as (4π × *convex hull area*) / (*convex hull perimeter*)^2.

12. ***Diameter of the bounding circle*** is the diameter (expressed in µm) of the smallest circle that encloses the convex hull.

13. ***Maximum span across the convex hull*** is the maximum distance between two points across the convex hull.

14. The ***ratio maximum/minimum convex hull radii*** is the division of the largest to the smallest radius from the center of mass of the convex hull to an exterior point.

15. The ***mean radius*** was calculated as the mean length in microns from the center of mass of the convex hull to an exterior point.

FracLac for ImageJ: https://imagej.nih.gov/ij/plugins/fraclac/FLHelp/Introduction.htm

Step guide to perform the morphological analysis of microglial cells using FIJI

Each study needs a specific approach based on its hypothesis and objectives. The steps taken in this study have been carefully chosen after empirical testing. The analysis was performed using FIJI software (freely downloadable from http://fiji.sc/Fiji). This analysis was done on binarized images of individual microglial cells. For this purpose, each image obtained with the confocal microscope was processed as follows:

1. To separate the green channel (Alexa Fluor 488, corresponding to the microglial marker Iba1) from the red channel (Alexa Fluor 594, corresponding to the inflammation marker IL-1β): Image → Color → Split channels.
2. The resulting image named C1 (corresponding to the green channel =Iba1) was selected, and the layers forming the z-stack were grouped: Image → Stacks → Tools → Grouped z Project; at this point, the layers selected for grouping were “all layers”, and the intensity selected was “maximum”.
3. The image was then binarized: Process → Binary → Make Binary.
4. The binarized image was manually edited to provide a cell profile formed by a single set of pixels. To ensure the homogeneity of criteria, this editing task was carried out by the same person. This edition consisted on: 1), removing the extensions of neighboring cells, and 2) connecting the cellular processes which resulted broken after binarization. To do this editing in an accurate way, the original image of the cell was used as reference. The elements to be removed were selected by polygon selections (rectangular box) and then cleared (Edit → clear). The manual connection of any two parts of the image was done by using the free-hands line (segment box) and later filling it (Edit → fill). The images had a white foreground (cell profile) and a black background. These colors were inverted if necessary (Edit → invert). Once processed, the image was centered: the cell was framed in the rectangle (rectangular box) and cut out (Image → crop).

5. The binarized and edited image of each microglial cell was named and saved as a .*Tiff* file. The name of each file allowed the tracking of each microglial cell for the subsequent steps of the morphological analysis. These type of files were the “Fill” images. All the cell images were placed in the same folder: the “Fill Folder”.
6. In parallel, a second image of each microglial cell, consisting in the profile of the cell, was created (Process → Binary → Outline). These new images were saved in a new folder: the “Outline Folder”.
7. These two sets of image files were used to measure specific parameters. “Fill” images were used to quantify *lacunarity* and *cell area*. Besides, *fractal dimension* and *cell perimeter* were measured from “Outline” images. The parameters based on the *convex hull* were obtained preferably from “Fill” images. The parameters *cell circularity*, *convex hull circularity*, *density* and *roughness* were calculated afterwards from the previously measured parameters.

Measurement of morphological parameters on microglial images by FracLac plugin

The Glossary for the FracLac Manual for morphological analysis is available at the page https://imagej.nih.gov/ij/plugins/fraclac/FLHelp/Glossary.htm.

The tools/methodology selected need to be defined for each particular study. It is strongly advisable to read the explanation of each tool/methodology provided by FracLac in order to choose the appropriate set. The steps followed in this work were:

1. FracLac was opened: Plugins → Fractal analysis → Fraclac. Then, BC box (Box Counting) was selected without selecting “Legacy model”.
2. The boxes selected were “Use binary” and “Lock black as background”.
3. In “Grid design”, “Power series” with base 2 and exponent 2 were selected.
4. In Files, “results” was selected.
5. And finally, in Graphics options, “Hull and circle → metrics”, “bounding circle” and “convex hull” were selected.
6. After clicking “OK” a new dialog box opens, where Db (to measure the fractal dimension by box counting) was chosen.

Once defined the tools for fractal analysis, the folder containing the images to analyze has to be selected as follows: after clicking “Batch” a dialog box appear to search for and select the folder of “Fill” or “Outlined” images. A new dialog box asks where to save the results. It is important to select the appropriate image folder for the parameter to be measured. Two “results” files were generated, which were opened from Excell and tabulated:

- the “Box count sumary” file, showed the *lacunarity* and the *fractal dimension;*

- the “Hull and circle results” file, contained the values of other morphological parameters.
