# Supplementary figures and images for: Microglial Morphometric Parameters Correlate With the Expression Level of IL-1β, and Allow Identifying Different Activated Morphotypes
Source: Front Cell Neurosci. 2019 Oct 25;13:472. doi: 10.3389/fncel.2019.00472 (PMC6824358; doi:10.3389/fncel.2019.00472)

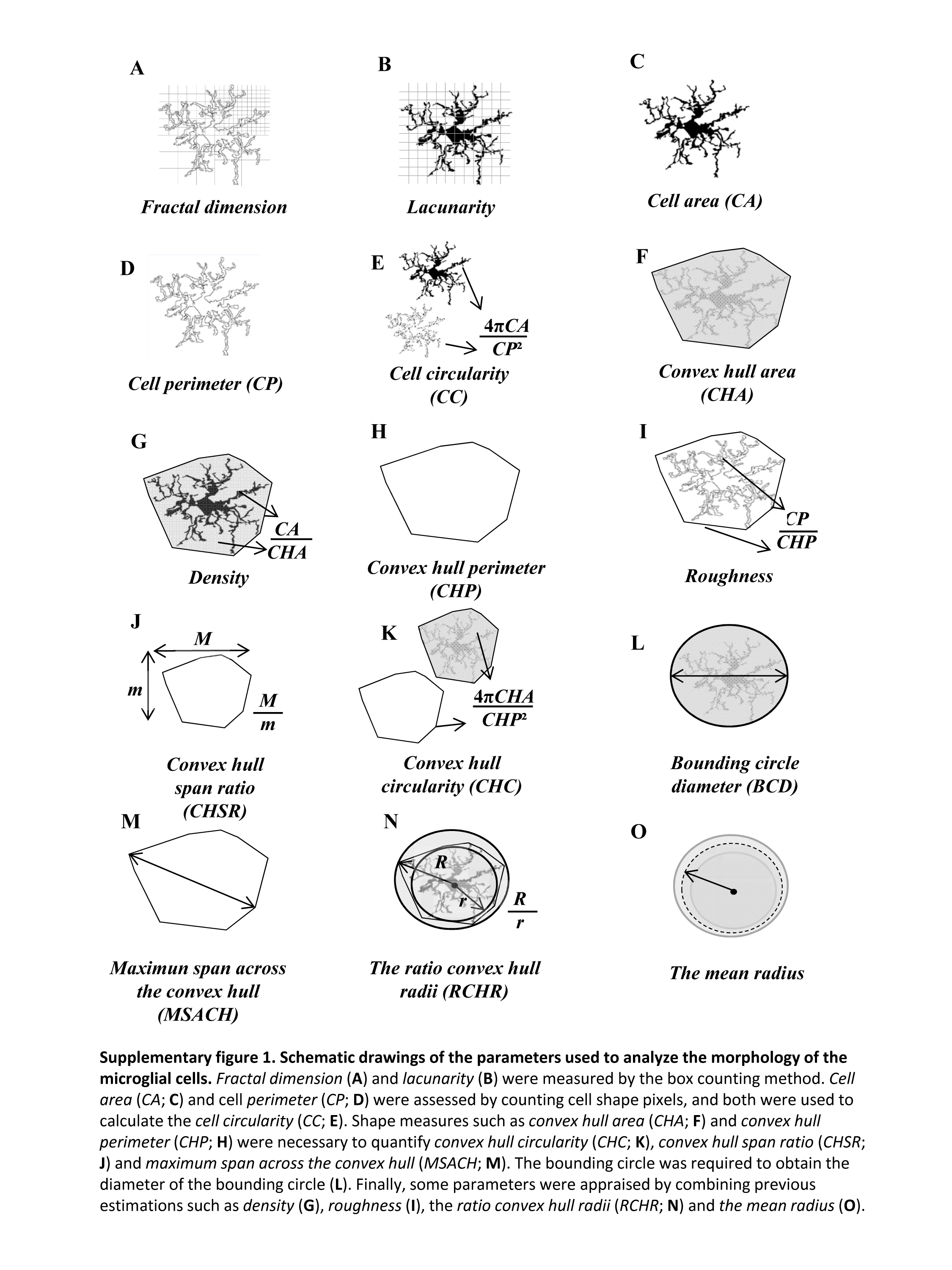

Supplement: Supplementary file 2 [file Image_1.JPEG]

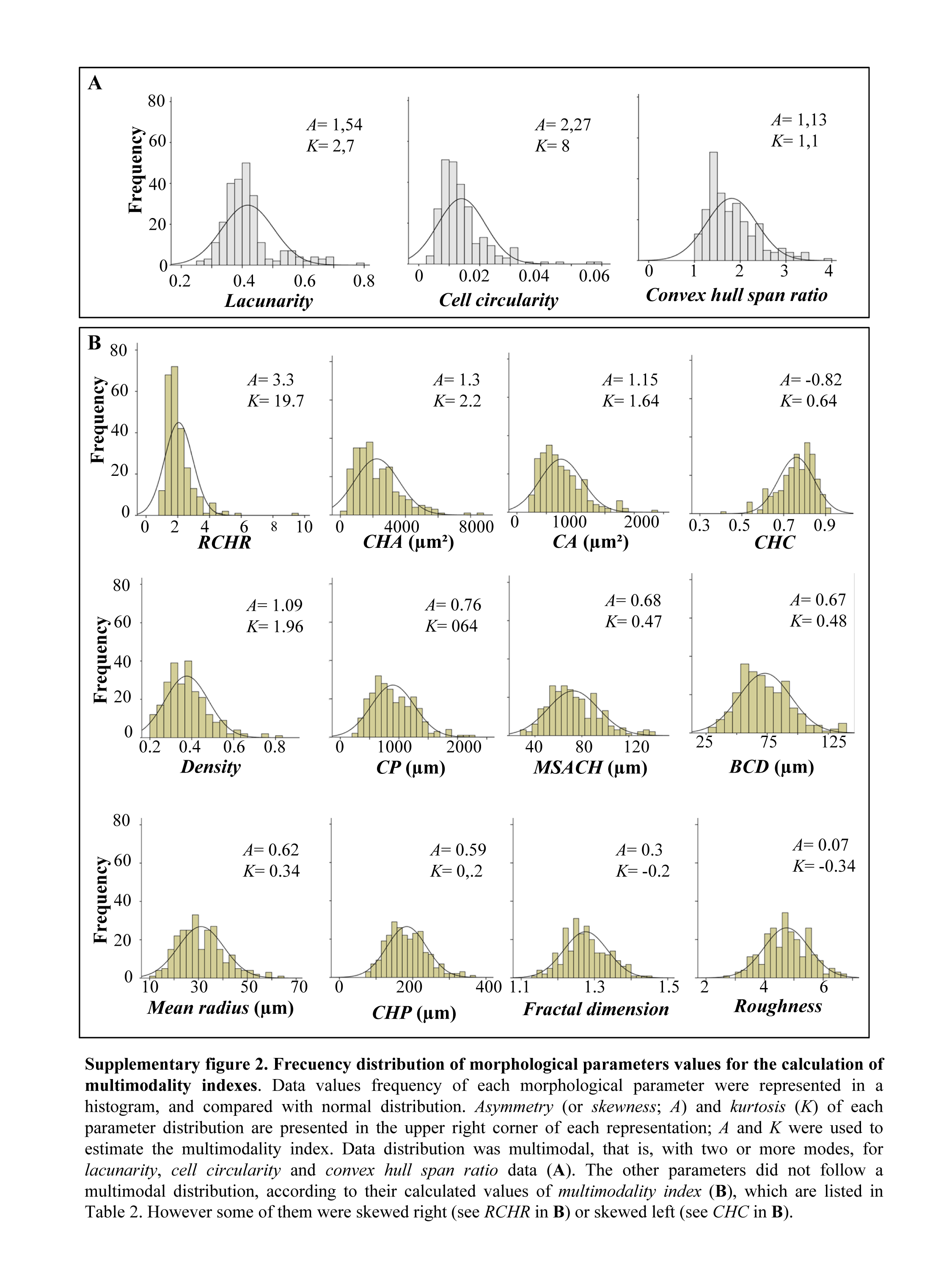

Supplement: Supplementary file 3 [file Image_2.JPEG]

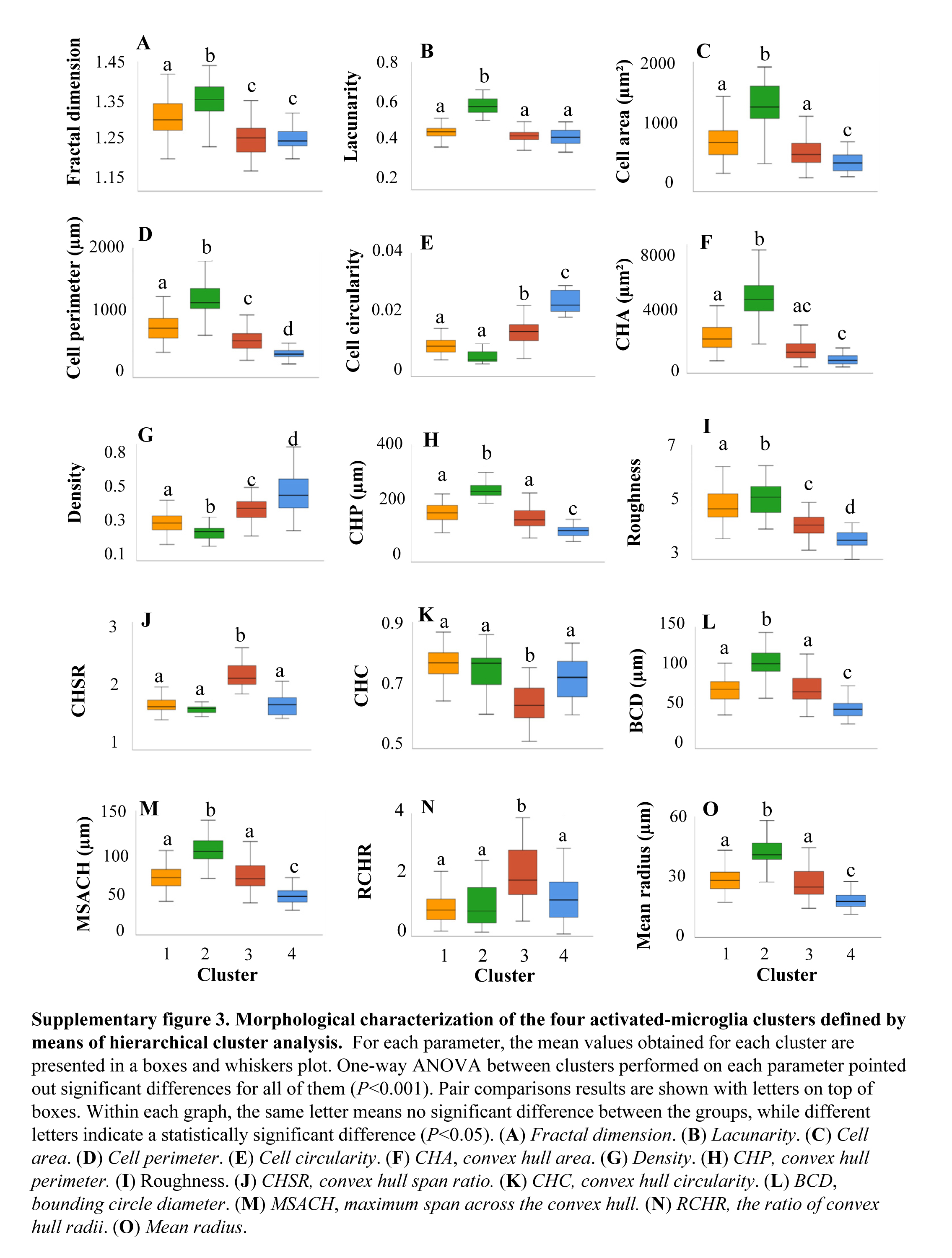

Supplement: Supplementary file 4 [file Image_3.jpg]
